# Acute febrile illness is associated with *Rickettsia* spp infection in dogs

**DOI:** 10.1186/s13071-015-0824-3

**Published:** 2015-04-10

**Authors:** Laia Solano-Gallego, Alessandra Caprì, Maria Grazia Pennisi, Marco Caldin, Tommaso Furlanello, Michele Trotta

**Affiliations:** Departament de Medicina i Cirurgia Animals, Facultat de Veterinària, Universitat Autònoma de Barcelona (UAB), Cerdanyola, 08193, Barcelona Spain; Royal Veterinary College, University of London, Hatfield, United Kingdom; Clinica Veterinaria Camagna, Reggio, Calabria Italy; Dip.to di Scienze Veterinarie, Polo Universitario Annunziata, Messina, Italy; Laboratorio Veterinario San Marco, Padova, Italy

**Keywords:** Dog, Febrile illness, *Rickettsia conorii*, *Ehrlichia*, *Anaplasma*, *Babesia*, *Hepatozoon*, *Leishmania*, Serology, PCR

## Abstract

**Background:**

*Rickettsia conorii* is transmitted by *Rhipicephalus sanguineus* ticks and causes Mediterranean Spotted Fever (MSF) in humans. Although dogs are considered the natural host of the vector, the clinical and epidemiological significance of *R. conorii* infection in dogs remains unclear. The aim of this prospective study was to investigate whether *Rickettsia* infection causes febrile illness in dogs living in areas endemic for human MSF.

**Methods:**

Dogs from southern Italy with acute fever (n = 99) were compared with case–control dogs with normal body temperatures (n = 72). Serology and real-time PCR were performed for *Rickettsia* spp., *Ehrlichia canis*, *Anaplasma phagocytophilum/A. platys* and *Leishmania infantum.* Conventional PCR was performed for *Babesia* spp. and *Hepatozoon* spp. Acute and convalescent antibodies to *R. conorii, E. canis* and *A. phagocytophilum* were determined.

**Results:**

The seroprevalence rates at first visit for *R. conorii*, *E. canis*, *A. phagocytophilum* and *L. infantum* were 44.8%, 48.5%, 37.8% and 17.6%, respectively. The seroconversion rates for *R. conorii*, *E. canis* and *A. phagocytophilum* were 20.7%, 14.3% and 8.8%, respectively. The molecular positive rates at first visit for *Rickettsia* spp., *E. canis*, *A. phagocytophilum*, *A. platys*, *L. infantum*, *Babesia* spp. and *Hepatozoon* spp. were 1.8%, 4.1%, 0%, 2.3%, 11.1%, 2.3% and 0.6%, respectively. Positive PCR for *E. canis* (7%), *Rickettsia* spp. (3%), *Babesia* spp. (4.0%) and *Hepatozoon* spp. (1.0%) were found only in febrile dogs. The DNA sequences obtained from *Rickettsia* and *Babesia* PCRs positive samples were 100% identical to the *R. conorii* and *Babesia vogeli* sequences in GenBank®, respectively. Febrile illness was statistically associated with acute and convalescent positive *R. conorii* antibodies, seroconversion to *R. conorii*, *E. canis* positive PCR, and positivity to any tick pathogen PCRs. Fourteen febrile dogs (31.8%) were diagnosed with *Rickettsia* spp. infection based on seroconversion and/or PCR while only six afebrile dogs (12.5%) seroconverted (*P* = 0.0248). The most common clinical findings of dogs with *Rickettsia* infection diagnosed by seroconversion and/or PCR were fever, myalgia, lameness, elevation of C-reactive protein, thrombocytopenia and hypoalbuminemia.

**Conclusions:**

This study demonstrates acute febrile illness associated with *Rickettsia* infection in dogs living in endemic areas of human MSF based on seroconversion alone or in combination with PCR.

## Background

*Rickettsia conorii* is a Spotted Fever Group (SFG) *Rickettsia* that causes Mediterranean Spotted Fever (MSF) in humans in Mediterranean countries and Sub-Saharan Africa. The onset of MSF is abrupt and typical human cases present with high fever, flu-like symptoms, a black eschar at the tick bite site and a maculo-papular rash. Severe forms of the disease may present with major neurological manifestations and multi-organ involvement may occur. The mortality rate is estimated at around 2.5% and classical risk factors for severe forms include elderly age, cirrhosis, chronic alcoholism and glucose-6-phosphate dehydrogenase deficiency [1,2]. In Italy, more than 1000 human cases of MSF are reported annually mainly in Sicily, Sardinia, Lazio and Liguria [2,3]. Moreover, a more pathogenic subspecies of *R. conorii*, *R. conorii israelensis*, has been described in human cases in Sicily [4]. In addition, *R. conorii* Indian tick typhus strain has also been diagnosed in human patients in Sicily [5].

The organism is transmitted in the Mediterranean basin by the brown dog tick *Rhipicephalus sanguineus*. Mediterranean spotted fever is a human seasonal disease that is frequently diagnosed between April and October with a maximum peak in June-August [2] and it is well correlated with the presence of *Rh. sanguineus*. Ticks are possibly the main reservoir of *R. conorii* infection due to the existence of both transtadial and transovarial transmission and cofeeding in this tick sp. [1,2,6]. The role of dogs in maintaining zoonotic foci remains unclear [1]. Although dogs are the natural host of the vector, there is limited information on their susceptibility to Rickettsiae infection and on the development of long duration rickettsiemia able to allow transmission to a feeding vector tick [7]. However, experimental infections suggest that dogs can be competent reservoirs for *R. conorii* [6,7]. Due to high levels of *Rh. sanguineus* exposure, dogs have been used in epidemiological studies as sentinels for human MSF [8] and proximity to seroreactive dogs has been found as a risk factor for MSF in humans [9]. Several studies have reported anti-*R. conorii* antibodies by the indirect fluorescent antibody test (IFA) in dogs in *R. conorii* endemic countries such as Italy with seroprevalence rates ranging from 15.5% to 74% [10]. The very high seroprevalences detected in Italian dogs would suggest frequent exposure to *Rickettsia* spp. or persistent low-grade infection with a rickettsial organism or organisms that cross react with *R. conorii* antigens by IFA testing.

Studies reported the detection of *Rickettsia* DNA in the blood of Spanish and Italian dogs [11-14]. However, evidence that natural *R. conorii* infection causes illness in dogs remains limited. Illness has been associated with *R. conorii* natural infection in only a few dogs since human MSF was described in 1910 [15]. The possibility that *R. conorii* may cause a clinical disease in dogs is supported by the evidence of seroconversion in three sick dogs from Israel [16] and the association between anemia and seroreactivity to *R. conorii* antigens [17]. In addition, a study reported the association between male dogs and seroreactivity to *R. conorii* antigens as found in humans where males have higher rates of infection [2,17]. Moreover, febrile illness has been associated with *R. conorii* infection in three dogs from Sicily by means of seroconversion and PCR [13] and in seven dogs from Portugal by PCR [18]. For this reason, the clinical significance of infection with *R. conorii*, or other SFG rickettsiae in dogs, remains unclear and requires further characterization.

The aims of this prospective case–control study were: 1) to investigate the presence of *Rickettsia* spp. infection in dogs from southern Italy (Sicily and Reggio Calabria) with fever by means of serological and molecular tests when compared with case control dogs with normal body temperatures; 2) to evaluate whether *Rickettsia* spp. infection causes clinical disease and clinicopathological abnormalities by clinical history, physical examination and baseline laboratory tests; and 3) to evaluate co-infections with other microorganisms that cause canine vector borne diseases such as *Leishmania infantum*, *Ehrlichia* spp., *Anaplasma* spp., *Babesia* spp. and *Hepatozoon canis* and their clinical importance in sick dogs.

## Methods

### Dogs

The study was carried out between August-November 2009 and between April-November 2010 in Sicily, Italy. Dogs were enrolled in the study from 20 Veterinary clinics throughout Sicily (Catania, Caltanissetta, Enna, Messina, Palermo, Siracusa and Trapani) and from the south of Calabria (Reggio Calabria).

Physical and laboratory examinations were performed during the study at the time of onset of clinical signs and after one month or later. MSF is most commonly described in humans with a maximum peak in June and August [2]. Clinical questionnaires were distributed and filled by veterinarians to obtain detailed clinical data at the time of onset of clinical signs and after one month or later. Information collected from the questionnaires included the date of sample collection, age, sex, breed, type of housing (indoor *versus* outdoor), flea and tick exposure, presence of vectors on clinical examination, vaccination status, presenting complaint, clinical signs observed, previous medical history, clinical response to specific therapy and clinical status of the dog’s owner.

Blood serum samples were taken two times (first and second visit) when possible with an interval between samplings of one month in the majority of dogs or two months in few dogs, in order to evaluate acute (first visit) and convalescent (second visit) antibodies and seroconversion. Blood was taken in EDTA tubes at the time of diagnosis prior to doxycycline treatment and after one month or more. One hundred and seventy-one dogs were included in the study and they were divided into two groups: febrile dogs and afebrile dogs.

#### Febrile dogs

Ninety-nine dogs with febrile illness were enrolled in the study. The inclusion criteria were: 1) Dogs with an acute onset (less than 10 days of duration) of fever (≥39.5°C) were included. 2) Only dogs with informed consent signed by the owner were enrolled. The exclusion criteria were: 1) Dogs with an acute onset of fever and evidence of another disease involved such as heat-stroke, neoplasia, bacterial abscesses and pyometra; 2) Dogs with a recent history of doxycycline administration during the past two months; 3) Dogs without signed informed consent by the owner.

#### Matched case afebrile dogs

A case control set of dogs (n = 72) with no apparent disease or other diseases unrelated to febrile illness was randomly selected from dogs seen at the same veterinary clinics during the same month as the febrile dogs. The inclusion criteria were: 1) Healthy dogs evaluated for routine procedures such as vaccinations, elective surgery (neutering/spaying); 2) Sick dogs that did not present with acute fever (≥39.5°C) or suspicion of tick-transmitted disease; 3) Only dogs with informed consent signed by the owner were enrolled in the study. The exclusion criteria were: 1) Dogs recently treated with doxycycline during the last two months; 2) Dogs without signed informed consent by the owner.

Only privately owned dogs were included in the study and examined by routine non-invasive diagnostic procedures. Owners were accurately informed and an informed consent was signed by both owner and veterinarian. Eight dogs presented clinical signs but not fever.

Blood samples from dogs were taken for diagnostic purposes as part of clinical management and, therefore, no ethical approval was needed.

Unfortunately, only 92 out of 171 dogs enrolled in the study came back for a second visit a month or more after the first visit.

### Laboratory procedures

#### Routine laboratory tests

Complete blood count (CBC) with blood smear examination and platelet concentration, complete biochemical profile including C-reactive protein and serum electrophoresis were performed in all dogs at the time of diagnosis and second visit as previously described [19]. The samples were submitted in less than 24 hours from blood withdrawal to *Laboratorio Privato di Analisi Veterinarie San Marco* (Padua, Italy). Whole EDTA blood and serum samples for PCR and serology were stored at −20°C until used for molecular and serological testing.

#### Serological techniques (IFA or ELISA)

Indirect immunofluorescence assays (IFA) kits from Fuller Laboratories (Fullerton, CA, USA) were used for IgG antibodies detection against *R. conorii, E. canis* and *A. phagocytophilum* antigens following manufacturer’s instructions.

IFA does not distinguish between canine antibodies against *A. phagocytophilum* and *Anaplasma platys* [20]. In addition, the IFA test used for *Rickettsia conorii* is known to cross-react with other species of spotted fever group *Rickettsia* such as *R. rickettsii*, *R. helvetica*, *R. slovaca* and *R. massiliae* as stated in manufacturer’s instructions.

An antibody titer of 1:64 or greater was considered positive for *R. conorii* antigen. An antibody titer of 1:80 or greater was considered positive for *E. canis* and *A. phagocytophilum* antigens.

Seroconversion was defined as ≥ 2–4 fold increase convalescent antibody titers (second visit) when acute (first initial visit) titers were positive or as any positive antibody titers when acute (first initial visit) titers were negative. High antibody titers were defined as equal or greater of 1:512 (*R. conorii*) or 1:640 (*E. canis* and *A. phagocytophilum*). Paired samples were run the same day.

ELISA was performed to detect antibodies against *L. infantum* as previously described [21].

#### DNA extraction, real-time PCR for Rickettsia spp. and other infectious agents and DNA sequencing

DNA extraction was performed from EDTA-anticoagulated whole blood sample by the High Pure PCR Template Preparation Kit (Roche Applied Science, GmbH, Mannheim, Germany) in accordance with the manufacturer’s protocol with some modifications. Two hundred μL of blood were incubated with 40 μL of Proteinase K and with 200 μL of Binding Buffer at 72°C for 1 h. Subsequent steps were carried out according to the manufacturer’s instructions (Roche Applied Science, GmbH, Mannheim, Germany). DNA was eluted in 50 μL of elution buffer at 72°C [13].

A quantitative PCR (qPCR) for the detection of *Rickettsia* spp., *A. phagocytophilum/A. platys*, *E. canis* and *L. infantum* in DNA samples was performed. PCR amplifications were carried out for *Rickettsia* spp. [13,14], *E. canis*, *A. phagocytophilum/A. platys* (fragment of 16S rRNA gene) [22], and *L. infantum* (fragment of kinetoplast minicircle) [23] using LCSet primers and probes following manufacturer’s instructions (TIB MOLBIOL). Real-time PCR amplifications were carried out in Roche LightCycler, version 3.5.17, (Roche Diagnostic, GmbH, Mannheim, Germany), with a final volume of 10 μL, including 2.5 μL of the DNA template, 2 μL of Light Cycler Fast start reaction mix (FastStart DNA Master^PLUS^, Roche Diagnostic, GmbH, Mannheim, Germany), and 0.5 μL of LCSet primers and probes according to the manufacturer’s instructions (TIB Molbiol, Berlin, Germany). Amplifications were conducted in sealed 20 μL Light Cycler glass capillaries (Roche Diagnostic, GmbH, Mannheim, Germany). Thermal cycling for *Rickettsia* spp qPCR comprised an initial denaturation step at 95°C for 8 min, followed by 55 cycles of a denaturation at 95°C for 5 s, annealing at 59°C for 7 s, and extension at 72°C for 7 s, with quantification of fluorescence signal [13,14]. Thermal cycling for *A. phagocytophilum/A. platys* qPCR comprised an initial denaturation step at 95°C for 8 min, followed by 45 cycles of a denaturation at 95°C for 5 s, annealing at 61°C for 10 s, and extension at 72°C for 11 s, with quantification of fluorescence signal [22]. Thermal cycling for *E. canis* qPCR comprised an initial denaturation step at 95°C for 8 min, followed by 60 cycles of a denaturation at 95°C for 5 s, annealing at 61°C for 10 s, and extension at 72°C for 11 s, with quantification of fluorescence signal [22]. Thermal cycling for *Leishmania infantum* qPCR comprised an initial denaturation step at 37°C for 10 min, 95°C for 8 min, followed by 45 cycles of a denaturation at 95°C for 5 s, annealing at 60°C for 5 s, and extension at 72°C for 4 s, with quantification of fluorescence signal [23]. A real-time PCR reaction was considered positive when the normalized fluorescence signal [ratio of the signal from detection channel F2 (640 nm) to the signal from detection channel F1 (495 nm) at the end of the annealing step] showed an exponential increase in fluorescence. The melting temperature of probe–template hybrids was automatically determined by the analysis of the software, and was useful for species identification of *Rickettsia rickettsii versus* other *Rickettsia* species and *A. phagocytophilum versus A. platys. Rickettsia rickettsii* had a melting temperature of approximately 65°C, while *R. conorii* had a melting temperature of approximately 58.2°C. *Anaplasma phagocytophilum* had a melting temperature of approximately 66°C, while *A. platys* had a melting temperature of approximately 57°C. Each real-time PCR run include a blank, a negative control, and a DNA cloned amplicon as a positive control (LCSet positive control; TIB Molbiol, Berlin, Germany).

Conventional PCRs for the detection of fragment of 18S rRNA gene of *Babesia* spp. [24] and of 18S rRNA gene of *Hepatozoon* spp. [25] were also carried out, as previously described. The partial 18S rRNA gene for *Babesia* spp. was amplified by using conventional PCR analysis in a final volume of 50 μL, containing 2.5 μL of DNA template from each sample, 1 μL (10 pmol/ μl) of each of the primers, PIRO-A and PIRO-B, 1 μL (10 pmol/μl) of deoxyribonucleotide triphosphates (dNTPs) (Roche Diagnostics, GmbH, Mannheim, Germany), 5 μL of PCR buffer 10X (5 PRIME GmbH, Hamburg, Germany), and 1U of recombinant Taq DNA polymerase (5 PRIME GmbH, Hamburg, Germany). DNA amplification was performed with the Thermal Cycler 2720 (Applied Biosystems, Europe) at the following temperature profiles: initial denaturation at 95°C for 5 minutes, denaturation at 95°C for 30 seconds, annealing at 58°C for 30 seconds, extension at 72°C for 30 seconds, and repeated for 35 cycles, with a final extension for 7 minutes at 72°C. The partial 18S rRNA gene (666 bp) for *Hepatozoon canis* was amplified by using conventional PCR analysis in a final volume of 50 μL, containing 5 μL of DNA template from each sample, 1 μL (10 pmol/μl) of each of the primers, HEP-F and HEP-R, 1 μL (10 pmol/μl) of dNTPs (Roche Diagnostics, GmbH, Mannheim, Germany), 5 μL of PCR buffer 10X (5 PRIME GmbH, Hamburg, Germany), and 1 U of recombinant Taq DNA polymerase (5 PRIME GmbH, Hamburg, Germany). DNA amplification was performed with the Thermal Cycler 2720 (Applied Biosystems, Europe) at the following temperature profiles: initial denaturation at 95°C for 5 minutes, denaturation at 95°C for 30 seconds, annealing at 53°C for 30 seconds, extension at 72°C for 30 seconds, and repeated for 35 cycles, with a final extension for 7 minutes at 72°C.

DNA electrophoresis for amplicons detection was carried out in 2.2% FlashGel System (Lonza, Rockland, ME USA), and DNA fragments were visualized. For subsequent sequencing of *Babesia* spp. and *Hepatozoon* spp. positive samples, amplification product from PCR was submitted for direct sequencing.

*Rickettsia* positive samples by qPCR for 16S gene were also amplified for the OmpA gene by a conventional PCR for further molecular characterization [13,14]. The partial OmpA gene for *Rickettsia* spp was amplified by PCR in a final volume of 50 μL, containing 5 μL of DNA template from each sample, 1 μL (10 pmol/μl) of each of the primers, 107F and 299R 1 μL (10 pmol/μl) of dNTPs (Roche Diagnostics, GmbH, Mannheim, Germany), 5 μL of PCR buffer 10X (5 PRIME GmbH, Hamburg, Germany), and 1U of recombinant Taq DNA polymerase (5 PRIME GmbH, Hamburg, Germany). DNA amplification was performed with the Thermal Cycler 2720 (Applied Biosystems, Europe) at the following temperature profiles: initial denaturation at 95°C for 5 minutes, denaturation at 95°C for 30 seconds, annealing at 54°C for 30 seconds, extension at 72°C for 30 seconds, and repeated for 35 cycles, with a final extension for 7 minutes at 72°C.

The samples were sequenced and compared to known sequences deposited in GenBank®. The sequencing reaction was performed with the Applied Biosystem 3730xI DNA Analyzer on both strands by BMR Genomics srl (Padua, Italy) by using the dideoxychain-termination method [26]. Consensus sequences were aligned [(BIOEDIT version 7.0 (ClustalW)] with known sequences in GenBank® using the basic local alignment search tool (BLAST) available from (http://blast.ncbi.nlm.nih.gov/Blast.cgi?PROGRAM=blastn&PAGE_TYPE=BlastSearch&LINK_LOC=blasthome). Sequences were deposited in GenBank®.

Current *Rickettsia* infection was considered when dogs were positive to *Rickettsia* PCR and/or demonstrated seroconversion to *R. conorii* antigen.

### Statistical analysis

For univariate analysis, Chi-square and Fisher’s exact tests were used to test for associations between proportions and putative explanatory factors and univariate Odds ratios (OR) were calculated. The differences were considered significant if the *P*-value was <0.05. SPSS (IBM, USA) statistical software (version 19) was used for statistical analyses.

## Results

### Dogs

The dog population characteristics including breed, gender, age and presence or absence of ticks per each group are described in Table 1. Statistical associations were found between breed and presence or absence of ticks and febrile *versus* afebrile group (Table 1).

**Table 1:** **Description of dog population characteristics**

**DOGS**	**BREED**		**GENDER**		AGE*		**TICKS**	
	**N = 159**		**N = 171**		**n = 161**		**N = 121**	
	**Mixed**	**Pure**	**Male**	**Female**	**Young**	**Adult**	**Presence**	**Absence**
**Total**	58/159 (37.1%)	101/159 (63.5%)	94/171 (54.9%)	77/171 (45%)	16/161 (9.9%)	145/161 (90%)	31/121 (25.6%)	90/121 (74.3)
**Febrile**	40/58** (68.9%)	53/101 (52.4%)	40/94 (42.5%)	45/77 (58.4%)	13/16 (81.2%)	85/145 (58.6%)	29/31*** (93.5%)	46/90 (51.1%)
**Afebrile**	18/58 (31%)	48/101 (47.5%)	54/94 (57.4%)	32/77 (42.8%)	3/16 (18.7%)	60/145 (41.3%)	2/31 (6.4%)	44/90 (48.8%)

*For age classification, young was considered as less than 12 months of age and adult was considered as above 12 months of age **Chi-square = 4.126, *P* = 0.042 ***Chi-square = 17.622, *P* < 0.0001.

### Serological and PCR results for all pathogens

The serological results in febrile and afebrile dogs at the time of the first and second visits are shown in Table 2. The total first visit seropositivity rates for *R. conorii*, *E. canis*, *A. phagocytophilum* and *L. infantum* were 44.8%, 48.5%, 37.8% and 17.6%, respectively. The seroconversion results for *R. conorii*, *E. canis* and *A. phagocytophilum* antigens in both groups of dogs are displayed in Table 3. The total seroconversion rates for *R. conorii*, *E. canis* and *A. phagocytophilum* were 20.7%, 14.3% and 8.8%, respectively. Seroconversion rates for *R. conorii* were significantly higher in febrile dogs when compared with afebrile dogs (Chi-square = 4.070, *P* = 0.0436). The seroconversion rates for *E. canis* and *A. phagocytophilum* were not different among the groups studied (Table 3).

**Table 2:** **Seroreactivity to several tick-borne pathogens antigens in febrile and afebrile dogs at the time of first and second visit**

**Pathogens**	**Initial serology (first visit)**			**Convalescent serology (second visit)**		
	**Febrile**	**Afebrile**	**Total**	**Febrile**	**Afebrile**	**Total**
	**N=95**	**N=70**	**N=165**	**N=44**	**N=48**	**N=92**
***R. conorii***	53 (55.8%)*	21 (30.0%)	74 (44.8%)	29 (65.9%)**	15 (31.2%)	44 (47.8%)
***E. canis***	47 (50.0%)	32 (46.4%)	79 (48.5%)	15 (34.1%)	11 (22.9%)	26 (28.2%)
***A. phagocytophilum***	38 (40.4%)	24 (34.3%)	62 (37.8%)	9 (20.4%)	7 (14.6%)	16 (17.4%)
***L. infantum*** ***	20 (20.2%)	10 (14.1%)	30 (17.6%)	Not determined		

*Chi-square = 10.837, *P* = 0.001;**Chi-square = 11.052, *P* = 0.002; *** Number of febrile dogs was 99; Number of afebrile dogs was 71; total number of dogs was 170.

**Table 3:** **Seroconversion results for** ***R. conorii*** **,** ***E. canis*** **,** ***A. phagocytophilum*** **in febrile and afebrile dogs**

**Pathogens**	**Seroconversion**		
	**Number of positive dogs (%, CI 95%)**		
	**Febrile**	**Afebrile**	**Total**
	**N = 44**	**N = 48**	**N = 92**
***R. conorii***	13 (29.5%, 18-44%)*	6 (12.5%, 5-25%)	19 (20.7%, 13-30%)
E. canis**	7 (16.3%, 7-29%)	6 (12.5%, 5-25%)	13 (14.3%, 8-22%)
A. phagocytophilum**	3 (7.0%, 1-18%)	5 (10.4%, 4-22%)	8 (8.8%, 4-16%)

*Chi-square = 4.070, *P* = 0.0436;**Number of febrile dogs was 43; number of total dogs was 91.

The total first visit molecular positive rates for *Rickettsia* spp., *E. canis*, *A. phagocytophilum*, *A. platys*, *L. infantum*, *Babesia* spp. and *Hepatozoon* spp. were 1.8%, 4.1%, 0%, 2.3%, 11.1%, 2.3% and 0.6%, respectively. Positive PCR for *E. canis* (7.1%), *Rickettsia* spp. (3%), *Babesia* spp. (4%) and *Hepatozoon* spp. (1%) were only found in febrile dogs (Table 4).

**Table 4:** **Molecular prevalence of all pathogens in febrile and afebrile dogs at the first visit**

**Pathogens**	**PCR**		
	**Febrile**	**Afebrile**	**Total**
	**n = 99**	**n = 71**	**n = 170**
***R. conorii***	3 (3.0%)*	0 (0%)	3 (1.8%)
***E. canis***	7 (7.1%)**	0 (0%)	7 (4.1%)
***A. platys***	3 (3.0%)	1 (1.4%)	4 (2.3%)
***B. vogeli***	4 (4.0%)	0 (0%)	4 (2.3%)
***H. canis***	1 (1.0%)	0 (0%)	1 (0.6%)
***L. infantum***	14 (14.1%)	5 (6.9%)	19 (11.1%)

*All dogs were positive to 16SrRNA and OmpA PCRs; ** Fisher’s exact test, *P* = 0.042

Fourteen febrile dogs (31.8%) were diagnosed with *Rickettsia* spp. infection based on seroconversion and/or PCR while only six afebrile dogs (12.5%) seroconverted (Chi-square = 5.036; *P* = 0.0248; Fisher’s exact test; *P* = 0.0414).

Febrile illness was statistically significant associated with: acute *R conorii* positive antibody reaction (Chi-square = 10.837; univariate Odds ratio = 2.944; *P* = 0.001); convalescent *R. conorii* positive antibody reaction (Chi-square = 7.312; univariate Odds ratio = 4.253; *P* = 0.001); seroconversion to *R. conorii* (Chi- square = 4.070; univariate Odds ratio = 2.935; *P* = 0.044); *E. canis* positive PCR (Chi-square = 5.236; univariate Odds ratio = 1.247; *P* calculated with Fisher’s exact test = 0.042); *E. canis* or *Rickettsia* or *Anaplasma* PCRs positive (Chi-square = 6.835; univariate Odds ratio = 9.793; *P* = 0.009); positivity to any tick pathogen PCRs (Chi-square = 10.161; univariate odds ratio = 13.687; *P* = 0.001).

Twelve febrile dogs (12.6%) displayed initial high *R. conorii* antibody titers while only five afebrile dogs (7.1%) presented initial high *R. conorii* antibody titers (Chi-square = 1.314; *P* = 0.252). Eleven febrile dogs (25%) displayed convalescent high *R. conorii* antibody titers while only 4 afebrile dogs (8.3%) presented convalescent high *R. conorii* antibody titers (Chi-square = 2.860; *P* = 0.091).

Five febrile dogs (35.7%) displayed initial high *E. canis* antibody titers while none the afebrile dogs (0.0%) presented initial high *E. canis* antibody titers (Chi-square = 3.840; *P* = 0.050). Five out of seven febrile dogs (71.4%) diagnosed with *E. canis* infection by means of PCR were seronegative (n = 2) or presented a low positive antibody titer (1:80; n = 3) at the time of illness and seroconverted suggesting acute infection. Only two febrile dogs diagnosed by positive *E. canis* PCR presented an initial high antibody titer (1:1280).

Three out of three febrile dogs (100%) diagnosed with *A. platys* infection by means of PCR were seronegative or presented a low positive antibody titer (1:80) by *A. phagocytophilum* IFA at the time of illness and only one seroconverted suggesting acute infection. Only one febrile dog diagnosed by positive *A. platys* PCR presented high antibody titer (1:1280).

Initial *E. canis* IFA was statistically associated with *A. phagocytophilum* IFA (Chi-square = 93.490; *P* < 0.0001).

#### Co-infections

Seven febrile dogs diagnosed with *Rickettsia* spp. infection based on seroconversion and/or PCR presented coinfections with *A. platys* diagnosed by PCR (n = 2), or with *E. canis* diagnosed by seroconversion (n = 1) or with both *E. canis* and *A. phagocytophilum* diagnosed by seroconversion (n = 2) or with *Babesia* diagnosed by PCR (n = 2). Two afebrile dogs diagnosed with *Rickettsia* spp infection based on seroconversion presented coinfections with *E. canis* diagnosed by seroconversion (n = 1) and with both *E. canis* and *A. phagocytophilum* diagnosed by seroconversion (n = 1).

Only febrile dogs presented coinfections with more than one pathogen demonstrated by molecular analysis: one coinfected with *R. conorii* and *A. platys*, one with *E. canis* and *H. canis*; one with *L. infantum* and *E. canis*, one with *L. infantum* and *A. platys* and two coinfected with *L. infantum* and *B. vogeli*.

#### Clinical manifestations and clinicopathological abnormalities

The most common clinical signs and laboratory abnormalities of febrile dogs diagnosed with *Rickettsia* infection based on seroconversion and/or PCR were fever, lethargy, myalgia, lameness, elevation of C-reactive protein, hypoalbuminemia, thrombocytopenia and mild non-regenerative anemia. However, other less common clinical signs and clinicopathological abnormalities included orchitis, lymphadenomegaly, splenomegaly, abdominal pain/hunched posture, vomiting and diarrhea, hyperglobulinemia, elevation liver enzymes (ALP, AST) and mature neutrophilic leukocytosis.

Anemia was statistically associated with the presence of an initial high *R. conorii* antibody titer (Chi-square = 3.877; *P* = 0.049), positive serology for *L. infantum* (Chi-square = 4.229; *P* = 0.040), positive *E. canis* PCR (Fisher’s exact test; *P* = 0.048), with positivity for *E. canis* or *Rickettsia* or *Anaplasma* PCR (Chi-square = 4.229; *P* = 0.040) and with positivity for any of the pathogens studied by PCR (Chi-square = 4.561; *P* = 0.033). Thrombocytopenia was statistically associated with the presence of positive convalescent *R. conorii* antibody titer (Chi-square = 4.227; *P* = 0.040), with acute high *E. canis* antibody titer (Chi-square = 10.809; *P* = 0.001), with positive convalescent *E. canis* antibody titer (Chi-square = 4.463; *P* = 0.035). It was also significantly associated with positive acute *A. phagocytophilum* antibody titer (Fisher’s exact test; *P* = 0.005), with high convalescent *A. phagocytophilum* antibody titers (Fisher’s exact test; *P* = 0.037), with *E. canis* positive PCR (Fisher’s exact test; *P* = 0.002), with positivity for *E. canis* or *Rickettsia* or *Anaplasma* PCR (Chi-square = 9.888; *P* = 0.002) and with positivity for any of the pathogens studied by PCR (Chi-square = 14.457; *P* <0.0001). Hemoparasites were not found by blood smear examination in any of the dogs included in the study.

#### DNA sequences

The OmpA gene DNA sequences (170 bp) obtained from three *Rickettsia* positive PCR samples (GenBank® accession numbers KP896303, KP896304 and KP896305) were 100% identical to several *R. conorii* sequences present in GenBank® (e.g.: GenBank® accession number KJ433804 and KF245453). The above OmpA gene DNA sequences were only 95% identical to the *R. parkeri* sequences present in GenBank® (GenBank® accession number: KJ158741, KJ174528, JQ906784) which were the second BLAST match.

The 18SrRNA gene DNA sequence (412 bp) obtained from four *Babesia* spp. positive PCR samples (GenBank® accession numbers KP896299, KP896300, KP896301 and KP896302) were 100% identical to the *B. vogeli* sequences present in GenBank® (GenBank® accession number: HQ148663). The positive amplicon for *Hepatozoon* spp. PCR was not sequenced due to the low amount of DNA in the sample.

## Discussion

This study demonstrates acute febrile illness associated with natural *Rickettsia* infection in dogs living in endemic areas of human MSF by means of seroconversion alone and the combination of seroconversion and/or PCR in comparison to an afebrile control group. Since human MSF was first described in 1910 by Conor and Bunch, canine illness associated with naturally occurring *R. conorii* infection has been described in only a few dogs. *Rickettsia conorii* infection associated with febrile illness was described in three dogs from Sicily by means of seroconversion and PCR [13] and seven dogs from Portugal by serology and PCR [18]. In addition, this study found significantly higher acute and convalescent *R. conorii* antibody titers in febrile dogs when compared with afebrile-control dogs.

Spotted fever group *Rickettsia* infection have so far only been recognized as causing acute infections in humans and dogs [1,27]. Therefore, acute and convalescent antibodies and PCR at the time of diagnosis were employed as diagnostic tools in this study to allow the detection of canine *Rickettsia* spp. infection. Interestingly, the majority of febrile dogs were diagnosed based on seroconversion to *Rickettsia* antigen and only three febrile dogs were diagnosed by molecular analysis in agreement with human MSF diagnostic findings [1,28,29]. Molecular detection of *Rickettsia* spp. such as *R. conorii* in blood appears to be of low to moderate sensitivity in humans [1,29,30] and in dogs [14] as found also in this study. The discrepancy between serological and molecular testing is likely due to the fact that *Rickettsia* spp. circulate in blood in low numbers during the acute phase of infection [2,31] and are probably rapidly cleared from blood. Accordingly, experimental infections of dogs with *R. conorii* resulted in a short rickettsiemia of 2–10 days post-infection [6,7] which did not recur following immunosuppression [15].

Clinical signs and clinicopathological abnormalities detected in febrile dogs diagnosed with *Rickettsia* spp. infection based on seroconversion or positive PCR at initial examination were similar to abnormalities associated with SFG rickettsiosis in humans [2,3,32,33] and Rocky Mountain Spotted Fever rickettsiosis in dogs [34,35] and in experimental infections of dogs with *R. conorii* [6,7]. The majority of experimentally *R. conorii* infected dogs presented acute fever, anorexia and lethargy [6,7]. In addition, similar clinical signs and clinicopathological abnormalities were detected in three client-owned Yorkshire terriers living in Sicily [13] and in seven client-owned Portuguese dogs [18].

Due to the fact that the majority of febrile dogs with *Rickettsia* spp. infection were diagnosed by seroconversion and the detection of rickettsiemia was not successful by molecular techniques in the majority of dogs, the *Rickettsia* species infecting the majority of these dogs remains unclear. It is well known that serological cross-reaction exists between different species of *Rickettsia* [28] and, therefore, *R. conorii* might not be the only pathogen infecting these dogs. However, based on current knowledge, the most likely species infecting dogs is *R. conorii* due to the fact that, so far, only *R. conorii* has been diagnosed by molecular techniques in the Mediterranean region [13,18] in dogs. In addition, the most common *Rickettsia* pathogen recognized in Sicily in humans and ticks is *R. conorii* [36] including different strains [4,5]. Other *Rickettsia* species such as *R. slovaca* [5], *R. africae*, *R. aeschlimannii* [37] and a new spotted fever group *Rickettsia* [38] have been occasionally found in Sicily in ticks and *R. massiliae* has been isolated from one human patient [39]. Additionally, three febrile dogs from the present study were diagnosed by DNA amplification and sequencing of *R. conorii* as the most likely infecting pathogen. Moreover, the three previous canine rickettsiosis cases reported in Sicily were diagnosed as *R. conorii* infection as well [13]. However, the pathogenic role of *R. massiliae* might be more important than previously thought and this rickettsia might cause MSF in humans [39-41] and subclinical [6] or febrile illness in dogs [42]. This is due to the facts that a moderate proportion of *Rh. sanguineus* ticks are infected with this microorganism [42-46], human cases of this infection have occasionally been reported [39-41] and *R. massiliae* antibodies are present in dogs [42,47] and humans [48,49]. Further studies should try to improve diagnostic techniques to better characterize the species of *Rickettsia* infecting dogs in Sicily and in the Mediterranean basin.

The findings of *Rickettsia* seroconversion in afebrile control dogs including healthy dogs suggest that, in some dogs, *Rickettsia* infection is subclinical. This finding is in agreement with experimental infections [6,7] and previous literature where dogs have been considered only sentinels of this infection [50,51]. Clinical manifestations of *R. conorii* or *Rickettsia* spp. infections can vary from subclinical to commonly mild disease in humans and other mammals including dogs, as demonstrated in the present study [2].

Serological and molecular positive rates obtained for *E. canis*, *A. phagocytophilum*, *A. platys*, *B. vogeli* and *L. infantum* are similar to previous studies performed in southern Italy [52,53] and other Mediterranean areas [54]. In contrast, there is insufficient information on seroconversion of tick-transmitted pathogens in sick and healthy dogs in endemic areas. Interestingly, higher seroconversions were found with *R. conorii* than with *E. canis* and *A. phagocytophilum* and *R. conorii* seroconversion was the only infection significantly associated with febrile illness in this study.

In the present study, febrile illness was associated with several tick pathogens and detection of DNA of pathogens was mainly observed in febrile dogs. In addition, coinfections were only detected by molecular techniques in dogs with febrile illness. The pathogenic role of coinfections remains unclear and is scarcely investigated [55,56]. However, the findings of this study suggest that coinfections diagnosed by PCR demonstrating active infection might aggravate clinical manifestations as previously reported [55,57].

In this study, the majority of febrile cases were diagnosed as associated with acute *E. canis* infection based on PCR-positive and acute negative or low positive antibody titers and evidence of seroconversion. Interestingly, it is thought that the majority of clinical cases of *E. canis* infection correspond to the chronic phase of this disease [58-60] while, in our study, clinical signs and laboratory abnormalities were mainly found during acute infections. The association of antibody reactivity found between *E. canis* and *A. phagocytophilum* is not surprising due to the well-known cross-reaction that occurs between *Ehrlichia* and *Anaplasma* species [60]. In the present study, we consider that the *A. phagocytophilum* positive serological results are most likely due to *A. platys* infection in dogs rather than a true infection with *A. phagocytophilum* due to the fact that only *A. platys* was diagnosed by molecular techniques and the location of tested dogs in Sicily where only *A. platys* is prevalent in dogs [61].

There is limited clinical knowledge about *B. vogeli* infection in dogs in endemic areas. *Babesia vogeli* infection is considered to cause subclinical infection or a mild to moderate diseases in some dogs [24,62,63]. Surprisingly, in the present study, *B. vogeli* infection was only detected in febrile dogs while subclinical infection was not evident based on molecular analysis in this study. Unfortunately, *Babesia* serology and seroconversion were not performed as this could have decreased the number of truly infected dogs diagnosed in this study. Although blood molecular analysis is probably the most sensitive and accurate diagnostic technique for active *Babesia* infection in dogs [62], it is important to highlight that *Babesia* parasites were not observed on blood smear examination. Based on the present findings, *B. vogeli* should be included in the differential diagnosis of dogs with febrile illness and blood PCR appears to be the best diagnostic technique to use for its detection.

*Hepatozoon canis* infections in dogs are frequently subclinical in endemic areas and clinical cases are sporadically reported [64]. Interestingly, in the present study, only one young febrile dog was diagnosed with *H. canis* infection. These findings are unusual due to the fact that higher rates of *H. canis* infection are reported in Mediterranean endemic areas [54,64,65] and that subclinical infections were not found. In addition, the dog with hepatozoonosis was coinfected with an acute *E. canis* infection diagnosed by PCR. Unfortunately, the dog was lost during follow-up and clinical data about response to treatment was lacking. Whether the clinical signs and clinicopathological abnormalities observed were attributed only to *E. canis* infection or to both infections remains unclear and further studies would need to elucidate the role of coinfections and *H. canis* in febrile illness in endemic areas.

## Conclusions

In conclusion, this study demonstrates acute febrile illness associated with natural *Rickettsia* infection in dogs living in MSF endemic areas based on acute and convalescent antibody titers, seroconversion alone and the combination of seroconversion and PCR. The most common clinical signs and clinicopathological abnormalities were similar to those described in human and canine spotted fever rickettsiosis. Additional febrile illness was associated with *E. canis* infection demonstrated by PCR, and with detection of tick pathogens and co-infection demonstrated by molecular techniques.

## Abbreviations

DNA: Deoxyribonucleic acid
IFA: Immunofluorescence antibody assay
qPCR: Quantitative PCR
MSF: Mediterranean Spotted fever
PCR: Polymerase chain reaction
SFG: Spotted fever group
